# Long non-coding RNA LINC01296 acts as a migration and invasion promoter in head and neck squamous cell carcinoma and predicts poor prognosis

**DOI:** 10.1080/21655979.2021.1967033

**Published:** 2021-09-13

**Authors:** Jing Wu, Hua Chen, Jingjing Li, Xiaoyu Li, Jun Cao, Min Qi

**Affiliations:** a Department of Stomatology, The People’s Hospital of Longhua District, Shenzhen, China; b Department of Plastic and Cosmetic Surgery, Xiangya Hospital, Central South University, Changsha, China

**Keywords:** Long non-coding RNA, LINC01296, head and neck squamous cell carcinoma, migration, invasion, prognosis

## Abstract

Long non-coding RNA (lncRNAs) can participate in gene expression regulation. LINC01296 is abnormally expressed in different tumors and promotes tumorigenesis and development. However, the role of LINC01296 in head and neck squamous cell carcinoma (HNSCC) remains not entirely clear. Thus, to explore LINC01296 expression, biological function and potential mechanism in HNSCC, we used GEPIA and GEO database. QRT-PCR was used to detect the knockout efficiency by LINC01296 inhibition with siRNA. Transwell assay was used to detect the migration and invasion capacity of tumor cells. Then enrichment and immunophenotype correlation analyses were carried out to explore the LINC01296 mechanism in HNSCC. To investigate why LINC01296 was up-regulated in HNSCC, DNA methylation analysis was performed using the DiseaseMeth database. LINC01296 expression was notably up-regulated in HNSCC, which was associated with promoter hypomethylation. Also, it was positively correlated with the HNSCC pathological stage and patients with higher LINC01296 expression levels had a poor prognosis. LINC01296 silencing inhibits HNSCC cell migration and invasion. LINC01296 also participate in the HNSCC progression mainly through protein phosphorylation and microtubule-based process regulation. Overall, LINC01296 was highly expressed in HNSCC, promoted tumor cells’ migration and invasion, and might be a potential diagnostic and prognostic marker in HNSCC patients.

## Graphical Abstract

**Figure:**
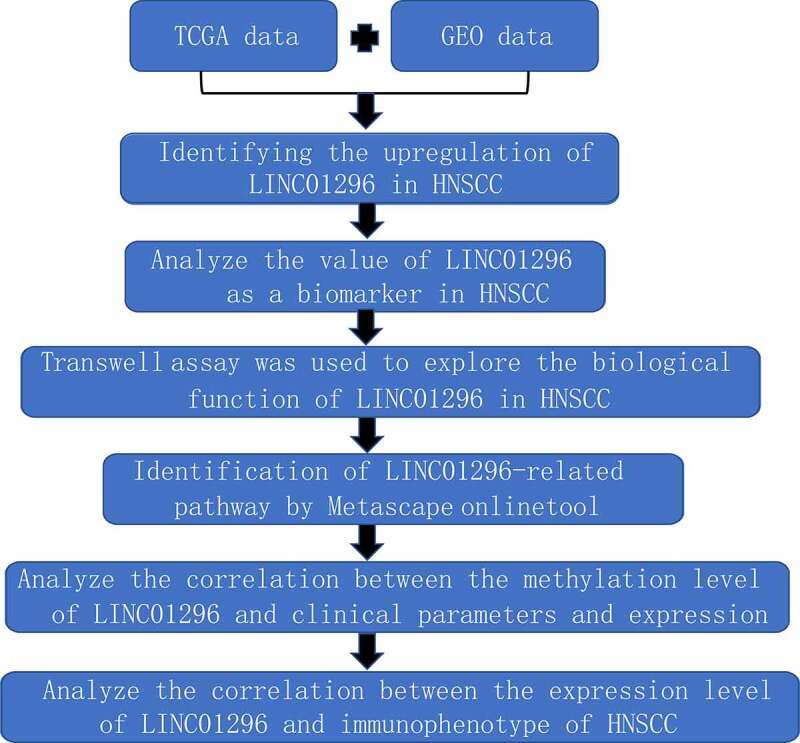

## Introduction

Head and neck carcinoma is one of the most common malignant tumors. It can vary with epidemiological factors and anatomical location, and has significant heterogeneity [1]. About 90% of its pathological types are squamous cell carcinomas, with strong invasion and high recurrence and metastasis rate, and a poor predicted clinical prognosis. Also, its local and advanced patient’s 5-year survival rates were about 69% and 34% respectively [2], and in 2018, it represented more than 800,000 new cases and more than 400,000 deaths worldwide [3]. Human papillomavirus (HPV) infection is one of its etiological factors, especially for the oral squamous cell carcinoma(OSCC) [4]. Moreover, about 75% of head and neck cancer are related to alcohol and tobacco [5–7], and genetic susceptibility, oral hygiene and occupational exposure may also be risk factors [7].

About 70%~90% of early-stage HNSCC patients have a longer survival time after surgical excision or radiation monotherapy [1]. Advanced patients require surgery, radiochemotherapy, and/or immunotherapy the combinations [8]. However, the recurrence rate is still around 50%, and may reach up to 65% in worse cases [1,9], and its median survival was only about 10 months [10]. Therefore, it is particularly important to explore the HNSCC occurrence and development mechanism, which will be beneficial to identify potential therapeutic targets and develop effective treatments, and finally improve the patients’ prognosis.

The development of high-throughput sequencing technology in the tumor area, allowed the exploration of non-coding RNAs’ (ncRNAs) biological functions [11]. An increasing number of long non-coding RNAs (lncRNAs) have been discovered, and might be used as potential therapeutic targets and diagnosis or prognosis biomarkers. LncRNAs are ncRNAs with more than 200 nt (nucleotides). They are mainly concentrated in the nucleus and do not encode proteins [12]. LncRNAs are important to the regulation of chromosome, growth, development and differentiation [13]. Moreover, they can be highly expressed in different cancers, including HNSCC [13], regulating tumorigenesis and cancer progression through various mechanisms. Up to now, the most studied lncRNAs include HOTAIR, HOTTIP, UCA1, LET, MEG3, MALAT1, H19 and NAG7 [12]. Therefore, exploring the role of different lncRNAs in HNSCC would provide new opportunities for better therapeutic strategies.

LINC01296–an intergenic lncRNA located on chromosome 14q11.2 [14]–can be highly expressed in gastric cancer, hepatocellular carcinoma, thyroid cancer, prostate cancer and other cancers, and can promote cancer progression [14–17]. On the other hand, LINC01296 can be down regulated in glioma and inhibit tumor proliferation, migration and invasion [18]. Altogether, these studies suggested that LINC01296 can present different expression levels in different tumor types, both as an oncogene or a tumor suppressor gene.

However, the LINC01296 biological characteristics and role in HNSCC have not yet been clarified. Therefore, we attempted to evaluate the LINC01296 expression, function and potential molecular mechanism in HNSCC. We hypothesized that LINC01296 served as an HNSCC oncogene and it might represent a promising target for HNSCC clinical treatment.

## Materials and methods

### Data mining

The LINC01296 expression data in HNSCC were analyzed with the GEPIA online database (44 normal and 519 tumors cases) [19]. We retrieved LINC01296 expression data in different pathological stages, subtypes and prognostics of HNSCC patients. Furthermore, we downloaded the mRNA expression datasets of HNSCC, nasopharyngeal carcinoma (NPC) and OSCC from the GEO database [20], then we used the three independent datasets (GSE29330, GSE12452 and GSE30784) to analyze LINC01296 differential expression.

### Cell culture & siRNA transfection

Human HNSCC cell lines (Cal-27 & SCC-9) were provided by the Department of Oral and Maxillofacial Surgery, Xiangya Hospital. Cal-27 cells were cultured in the Roswell Park Memorial Institute (RPMI)-1640 medium with 10% FBS, 1% penicillin and 1% streptomycin. The incubation conditions were: 5% CO_2_ and 95% humidity at 37 °C. SCC-9 cells were cultured in Dulbecco’s modified Eagle medium (DMEM) with 10% FBS,1% penicillin and 1% streptomycin. Incubator conditions were the same as the former. For gene silencing, cells were seeded and cultured overnight, then transfected with 50 nM LINC01296 siRNA (siRNA 1 and siRNA 2) or control (CTR) siRNA using Lipofectamine 3000 (Invitrogen, CA, USA) in the OptiMEM medium (Invitrogen). The siRNAs used in this study were synthesized by Sangon Biotech (Shanghai, China).

### RNA extraction & qRT-PCR

Total RNA was extracted from Cal-27 and SCC-9 cells according to the TRIzol reagent (Invitrogen) operation instructions. Then, they were reverse transcribed into cDNAs. qRT-PCR was accomplished by a First Strand cDNA Synthesis Kit (Roche, NJ, USA) in a Roche real-time PCR detection system (LightCycler480, Roche, USA). The primers used were: LINC01296, forward: 5´-AGTTCCACCAAGTTTCTTCA-3´ and reverse: 5´-AGGTTTTGCCAGCATAGAC-3´;β-actin(referencegene),forward:5´-TCACCAACTGGGACGACATG-3´and reverse: 5´-GTCACCGGAGTCCATCACGAT-3´.

### Transwell cell migration & invasion assays

This experiment was carried out according to procedures published in our previous research [21].

### Enrichment analysis

To understand the LINC01296 biological function in HNSCC cells, we used GEPIA2 to detect it’s similar genes (http://gepia2.cancer-pku.cn/#similar) [19]. Then, the Metascape website was used for functional enrichment analysis of these genes (https://metascape.org/gp/index.html#/) [22].

### Immunophenotype correlation analysis

Based on the TCGA data, we explored the relationship between LINC01296 (Alias: DUXAP9) and tumor neoantigen count, tumor mutation burden (TMB) and microsatellite instability (MSI). Additionally, we explored the relationship between LINC01296 and immune checkpoints and DNA damage repair genes in HNSCC. The above analyses were performed using the Sangerbox online tool (http://sangerbox.com/Signin).

### DNA methylation analysis

To further explore the LINC01296 overexpression causes in HNSCC, we downloaded the TCGA-HNSCC cohort DNA methylation data from the DiseaseMeth database (http://bio-bigdata.hrbmu.edu.cn/diseasemeth/) [23]. The prognosis and pathological data of those samples were retrieved from the UCSC Xena database (https://xena.ucsc.edu/) [24], and compared one by one.

### Statistical analysis

The data were analyzed with GraphPad Prism Software (CA, USA), the statistical results were analyzed as means ± standard error. Student’s t tests and analysis of variance were used to compare two groups and multiple comparisons, respectively. The Kaplan-Meier method was used to draw the survival curve and calculate the overall survival (OS). The log-rank test was used to compare the survival curves of the two groups. Log-rank *p*-value < 0.05 indicated that the difference was statistically significant.

## Results

LINC01296 can act as an oncogene or suppressor gene in different cancers, but it’s role in HNSCC is not clear. In this study, LINC01296 expression levels in HNSCC tissues increased and associated with patients’ prognoses. Transwell assays indicated that LINC01296 silencing suppresses cells migration and invasion. At the molecular level, our bioinformatics analysis results suggested that HTR2C, TACR3, MCHR2 and GHSR might be LINC01296 targets through tumor microenvironment and phosphorylation regulation. Additionally, DNA demethylation analysis suggested that LINC01296 overexpression was related to promoter hypomethylation.

### LINC01296 is highly expressed in HNSCC

**Figure 1.**
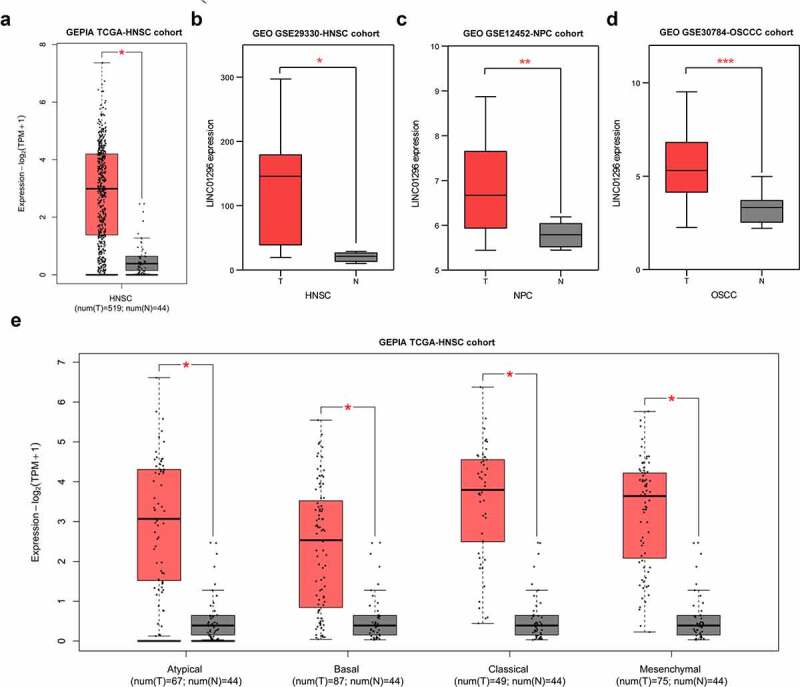
LINC01296 was upregulated in HNSCC

To assess LINC01296 expression levels in HNSCC, we used the GEPIA online tool to analyze the TCGA-HNSCC cohort, and HNSCC tissues were compared with the adjacent normal tissues. Results showed that LINC01296 expression was significantly higher in HNSCC tissues compared to normal ones (Figure 1(a)). Also, the expression in different subtypes showed us the same results (Figure 1(e)). Then, we use GSE29330, GSE12452 and GSE30784 datasets to explore LINC01296 gene differential expression (Table 1). Data analysis showed that the LINC01296 expression level was significantly increased than in normal tissues (Figure 1(b)), verifying the above result. In addition, LINC01296 expression was upregulated in NPC and OSCC tissues (Figure 1(c,d)).

**Table 1.** The means and standard deviations of LINC01296 expression levels in HNSCC based on three studies (from GEO database)

Study	Country	Year	Sample type	Type	Normal			Tumor		
					Number	Mean	SEM	Number	Mean	SEM
GSE29330	USA	2011	tissue	HNSCC	5	20.38	3.281	13	118.9	23.26
GSE12452	USA	2008	tissue	NPC	10	5.81	0.08325	31	6.853	0.1826
GSE30784	USA	2011	tissue	OSCC	45	3.241	0.1069	167	5.435	0.1268

SEM: standard error of mean; HNSCC: head and neck squamous cell carcinoma; NPC: nasopharyngeal carcinoma; OSCC: oral squamous cell carcinoma.

LINC01296 expression data in HNSCC was retrieved from the GEPIA online tool and GEO database. (1A&1B&E) LINC01296 was highly expressed in HNSCC tissues; (1 C&1D) LINC01296 was upregulated in NPC and OSCC tissues. *p < 0.01; ** *p*< 0.01;*** *p* < 0.001.

HNSCC: Head and neck squamous cell carcinoma; NPC: nasopharyngeal carcinoma; OSCC: oral squamous carcinoma; GEPIA: Gene Expression Profiling Interactive Analysis.

### The diagnostic and prognosis value of LINC01296 in HNSCC

**Figure 2.**
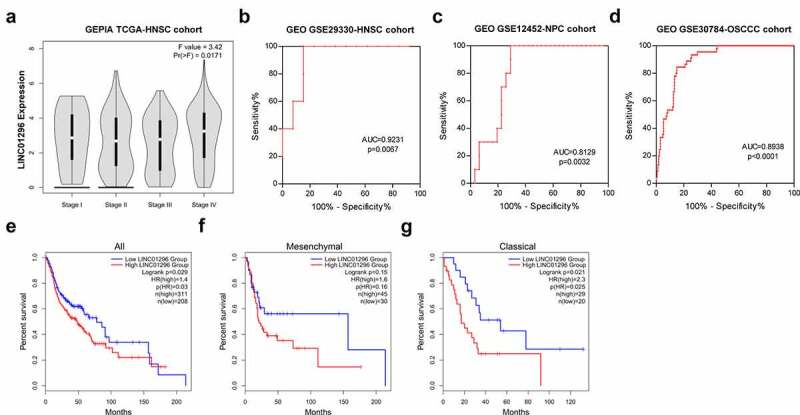
LINC01296 upregulation was associated with HNSCC patients’ poor prognosis, and has potential to be a HNSCC diagnostic marker

We investigated the association between LINC01296 expression levels and patients’ pathological stages. The data showed that the LINC01296 expression level was correlated with the pathological stage. The LINC01296 expression level was highest in stage, and was positively correlated with pathological grade except for stage (Figure 2(a)). We also collated the data in GSE29330-HNSCC, GSE12452-NPC and GSE30784-OSCC cohorts to analyze the LINC01296 diagnostic value in HNSCC, NPC and OSCC respectively. As shown in Figure 2(b-d), ROC curve indicated that LINC01296 is a highly accurate indicator for the diagnosis of HNSC, NPC and OSCC, especially for diagnosis of HNSC. In addition, the expression level was related to patients’ survival. Patients with higher LINC01296 expression had a poor prognosis in all HNSCC types (Figure 2(e)). The prognosis of high LINC01296 expression patients was significantly worse than those with low expression in the Classical HNSCC type (Figure 2(f)). The same result was for in the Mesenchymal type of HNSCC patients, however, there was no statistical significance (Figure 2(g)).

(a) The correlation between LINC01296 expression and patients’ pathological stage. (2B-D) LINC01296 has high accuracy as an HNSCC, NPC, and OSCC diagnostic indicator. (2E-G) HNSCC patients with higher LINC01296 expression had shorter overall survival time.

HR: Hazard rate; AUC: Area under the curve.

All: all HNSCC patients’ the subtypes of;Classical: Classical HNSCC patient type;Mesenchymal:Mesenchymal HNSCC patient type.

### LINC01296 silencing efficacy were measured by qRT-PCR

**Figure 3.**
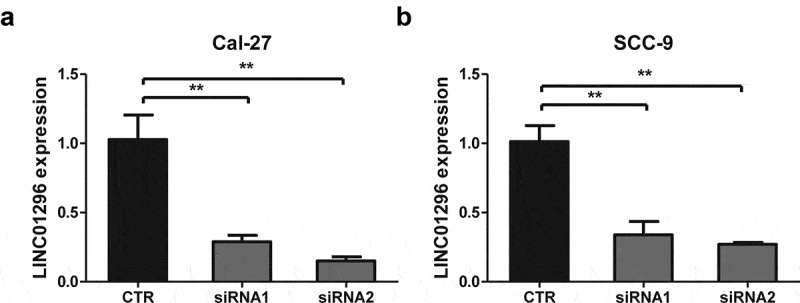
LINC01296 expression levels of in Cal-27 and SCC-9 cells by qRT-PCR after siRNAs transfections

To explore LINC01296 function in HNSCC, two siRNAs targeting LINC01296 were designed to silence endogenous genes for subsequent experiments. qRT-PCR analyses showed that both siRNA1 and siRNA2 had a silencing effect, but the siRNA2 efficacy was greater (Figure 3(a,b)). Therefore, the next experiments were performed using siRNA2.

The LINC01296 siRNA2 silencing efficacy was better than siRNA1 in both (a) Cal-27 and (b) SCC-9 cell lines. **p< 0.01.

CTR: Control; siRNAs (siRNA1 and siRNA2) targeting LINC01296;qRT-PCR: quantitative real-time PCR.

### LINC01296 gene silencing inhibits HNSCC cells migration and invasion

**Figure 4.**
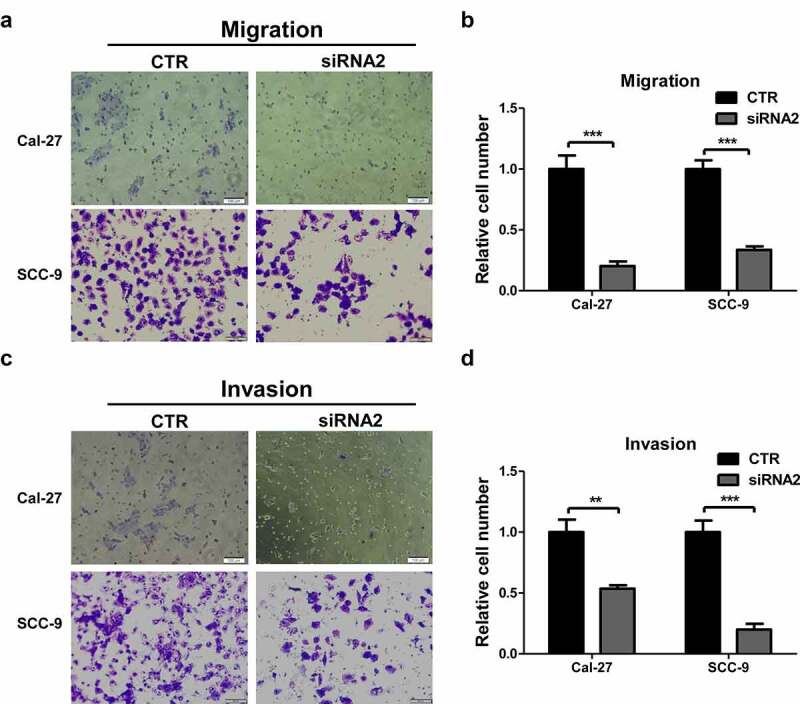
LINC01296 knockdown inhibits HNSCC cell migration and invasion *in vitro.*

The transwell assays were used to explore LINC01296 biological function in HNSCC. The in vitro experiments showed that the number of migrating cells in the LINC01296 siRNA group was significantly lower compared to CTR (Figure 4(a,b)). Cal-27 and SCC-9 cells showed consistent results. In addition, LINC01296 knockdown significantly decreased the invasion capacity of these cells (Figure 4(c,d)). These results confirmed that LINC01296 can promote HNSCC cells migration and invasion.

Cal-27 (a) and SCC-9 (b) LINC01296-deficient cell migration ability decreased compared to controls. Cal-27 (c) and SCC-9 (d) The of LINC01296-deficient cells invasion ability decreased compared to controls. **p< 0.01, ***p < 0.001.CTR: Control.

### Gene enrichment analysis

**Figure 5.**
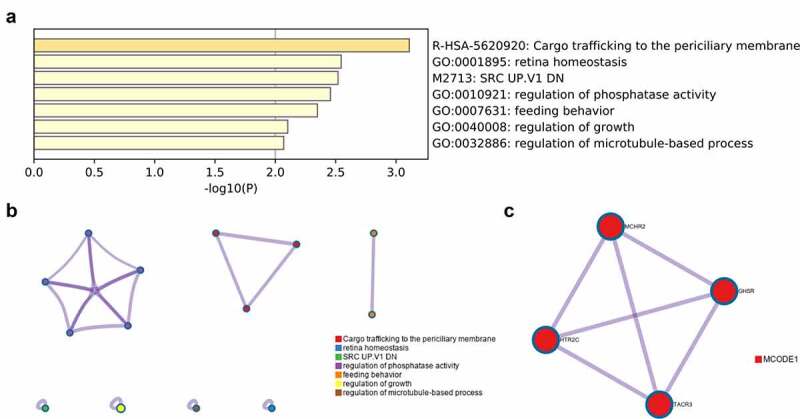
Function enrichment of similar genes expressed in HNSCC

To explore the possible LINC01296 molecular mechanism, we used the GEPIA database to find the top 300 genes with similar LINC01296 expression pattern. Then, we used the Metascape software for enrichment analysis. We found that 7 terms have the most significant enrichment: cargo trafficking to the periciliary membrane, retina homeostasis, SRC UP. V1 DN, regulation of phosphatase activity, feeding behavior, regulation of growth, regulation of microtubule-based process (Figure 5(a)). Further enrichment analysis on key modules was applied using the Mcode plug-in of the Metascape database. Three modules were significantly enriched: regulation of phosphatase activity, cargo trafficking to the periciliary membrane and feeding behavior (Figure 5(b)). Finally, the LINC01296 function in HNSCC may be closely related to four genes: HTR2C, TACR3, MCHR2 and GHSR (Figure 5(c)).

(a&b) Metascape analysis of the most significantly enriched similar genes functions. (c) The protein interaction network diagram shows the enrichment of four key genes.

### LINC01296 is associated with the HNSCC immunophenotype

**Figure 6.**
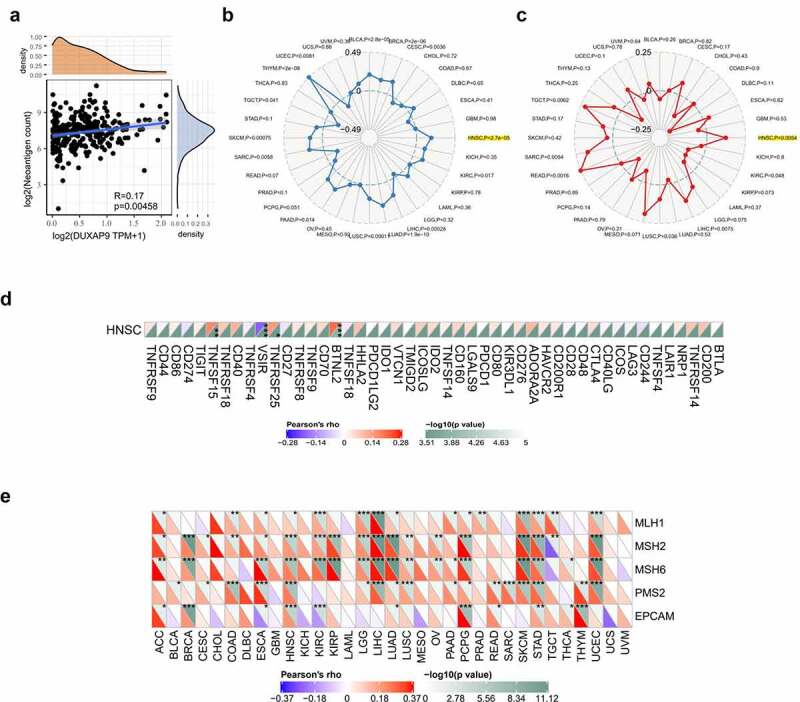
LINC01296 is associated with the HNSCC immunophenotype

LINC01296 was negatively correlated with the neoantigen count (Figure 6(a)). TMB and MSI analysis were better guide to clinical immunotherapy. The results indicated that LINC01296 expression was positively correlated with the relatively higher mutation and microsatellite instability status in HNSCC (Figure 6(b,c)). Figure 6(d) presents us that LINC01296 expression had a close link with immune checkpoint genes (BTNL2, VSIR, TNFSF15, TNFRSF25) in HNSCC. The results suggested that LINC01296 perhaps regulated tumor immune response by immune checkpoint regulation. In addition, LINC01296 expression in HNSCC was positively associated with the five MMR genes (MLH1, MSH2, MSH6, PMS2 and EPCAM), especially with MSH2, MSH6, PMS2 and EPCAM. That suggested that LINC01296 may play a role in tumors by regulating MMRs (Figure 6(e)).

(a)The correlation between LINC01296 (Alias: DUXAP9) and neoantigen count; (b&c) The relationship between LINC01296 expression and TMB, MSI in 33 cancers; (d)The relationship between LINC01296 and immune checkpoint genes in HNSCC; (e) The relationship between the LINC01296 expression of five important Mismatch repair (MMR) related genes (MLH1, MSH2, MSH6, PMS2 and EPCAM).*p< 0.05, **p< 0.01, ***p< 0.001.TMB: tumor mutation burden; MSI: Microsatellite Instability;

### DNA demethylation might explain LINC01296 up-regulation

**Figure 7.**
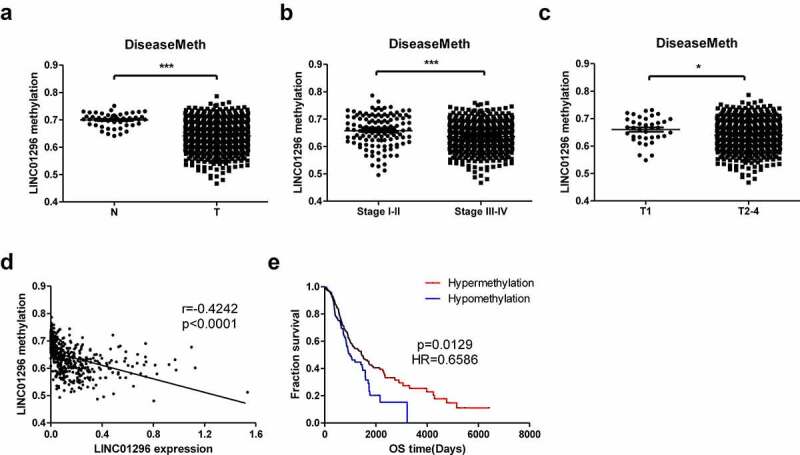
LINC01296 DNA hypomethylation in HNSCC

We also integrated and analyzed the methylation data of the TCGA HNSCC datasets retrieved from the DiseaseMeth database and the expression and clinical data of the TCGA HNSCC datasets from the UCSC Xena database. LINC01296 showed hypomethylation level in HNSCC tissues and relative hypermethylation status in paracancer tissues (Figure 7(a)). The methylation level was related to the HNSCC patients’ TNM stage. Also, the higher TNM stage (stage) had lower methylation levels compared to stage (Figure 7(b)). Additionally, the methylation level was correlated to tumor size or invasion depth, and the methylation levels in T2-4 were lower than in T1 (Figure 7(c)). The methylation level was negatively correlated with the LINC01296 expression (Figure 7(d)). Finally, patients in the hypermethylated state had a longer overall survival time and a better prognosis than those in the hypomethylated one (Figure 7(e)).

(a)The methylation level in tumor and normal tissues; (b&c) The methylation level in TNM and T stages; (d) Correlation between LINC01296 and gene methylation; (e) Hypermethylation HNSCC patients had a longer overall survival. *p< 0.05, ***p< 0.001.

## Discussion

Many studies have given evidence that lncRNAs are important for different biological functions and diseases occurrence and development, including tumors. Thus, lncRNAs, have gradually become a promising therapeutic target [25,26]. LINC01296, an intergenomic lncRNA located on chromosome 14 long arm, was discovered a few years ago [14]. As mentioned above, the LINC01296 expression has been up-regulated in a variety of tumors and was associated with patients’ prognoses. However, its biological characteristics in HNSCC are unknown.

Through data mining, we found that LINC01296 was also up-regulated in HNSCC, and was associated with a poor pathological stage. This result suggested that LINC01296 might be an oncogene in the HNSCC pathogenesis. In addition, LINC01296 had high diagnostic accuracy in HNSCC, the expression level of LINC01296 was associated with the HNSCC patients’ survival. Patients with high expression levels had shorter overall survival than those with low expression levels. Therefore, LINC01296 has potential as a diagnostic and prognostic HNSCC marker. Our study was consistent with the research of Wang K et al and Yu X et al [27,28]. This suggested that LINC01296 might be a prognostic pan-cancer marker, although it requires further validation in large samples.

Subsequent *in vitro* experiments showed that LINC01296 silencing could significantly inhibit the HNSCC cells’ migration and invasion, which showed the significance of further study LINC01296. These LINC01296 functions in tumor cells also were also observed in some other researches [16,29–32].

Phosphorylation is a protein post-translational modification that can be involved in pathophysiological events such as cell signal transduction, nerve activity, muscle contraction, cell proliferation and differentiation, and regulate cell growth, differentiation and apoptosis [33]. Increasing studies have shown that lncRNAs can bind to key signaling pathways’ signal mediators, such as protein kinases and receptors, and influence the signal cascade reaction process by kinase activity regulation. Its regulation of enzyme activity regulation mechanisms mainly include [34]: 1) Acting as a protein molecular chaperone: promotes enzyme conformational changes and regulates the activity through interaction with subunits; 2) Acting as an allosteric regulator: bind to the allosteric site to regulate activity; 3) Acting as a synergistic agent: bind a variety of enzymes and/or regulatory factors together to mediate effector or substrate synergistic effects; 4) LncRNAs can attach to the target protein surface, prevent the upstream signal pathway to recombine the target protein and prevent post-transcriptional modifications; 5) LncRNA can mediate the repositioning of binding proteins as a transporter. Wang J et al. found that the LOC441461 carcinogenic role of in colorectal cancer development was related to MLC and LIMK1 phosphorylation of via RhoA/ROCK signaling pathway [35].The lncRNA, NBR2, increased AMPK kinase activity under stress conditions, affected protein expression in tumor cells, and promoted tumor development [36]. LINC01296 can participate in tumor development by regulating phosphorylation. Zhang L et al. showed that LINC01296 can regulate liver cancer growth through Mir-26a /PTEN signaling pathway [16]. Wang ZL et al showed that the LINC01296/ Mir-143-3p /MSI2 axis regulated thyroid cancer development of through the AKT/STAT3 signaling pathway [15]. Additionally, researches have shown that LINC01296 can regulate MUC1 through the PI3K/AKT pathway and is involved in colorectal cancer progression [37]. Our study showed for the first time that the LINC01296 is important in HNSCC, but the specific mechanism remains to be further studied.

The tumor microenvironment is a complex environment in which tumor cells live. It is mainly composed of different extracellular matrices and stromal cells. Tumor microenvironment components can interact with tumor cells to promote tumor development and progression [38]. Ayano Kondo et al. showed that the lncRNA-JHDM1D-AS1 was up-regulated under nutrient starvation in the tumor microenvironment by regulating angiogenic factors expression and triggering inflammation around the tumor microenvironment, thus accelerating tumor growth and promoting malignancy [39]. Based on our enrichment and correlation analyses in our study, we found that the LINC01296 function might be closely related to ‘HTR2C, TACR3, MCHR2 and GHSR’ genes, and to immune checkpoint genes, BTNL2, VSIR, TNFSF15 and TNFRSF25. Therefore, we hypothesized that LINC01296 can affect immune cells’ function to regulate the behavior of tumor cells, influencing tumor microenvironment, and finally participating in tumor progression. This provided a direction and foundation for further researches. These factors may also contribute to provide directions for therapeutic strategies that combine targeted inhibitors with immune checkpoint blockade. However, the specific signaling pathways involed remains to be further studied.

DNA methylation is a common epigenetic gene expression regulation mechanism. Our methylation analysis suggested that LINC01296 up-regulation might be caused by hypomethylation in its promoter region. In addition, dynamic changes in DNA methylation level are associated with tumor cells’ development, and hypermethylation patients had better prognoses. Therefore, the data indicated that not only the LINC01296 expression level but also the methylation level of its promoter region can act as HNSCC prognostic markers.

## Limitation

Our study also has limitations such as the absence of *in vitro* experiments to validate downstream targets and explore LINC01296 the exact molecular mechanism in HNSCC. Moreover, our findings were not validated in animal experiments. Hence, additional studies are required to confirm our findings.

## Conclusion & future perspective

Overall, our study reported for the first time that LINC01296 is upregulated in HNSCC. Moreover, LINC01296 overexpression can promote HNSCC cells migration and invasion. This study lays a good foundation to our further researches. Further studies on mechanisms and pathways are necessary to verify the LINC01296 potential as a HNSCC prognostic marker. Altogether, these will promote the development of new HNSCC prognostic markers and therapeutic targets.

## Data Availability

The data supporting the findings reported in this study are available from the corresponding author upon reasonable request.

## Highlights

1. LINC01296 expression was upregulated in HNSCC tissues.
2. The LINC01296 upregulation was associated with HNSCC patients’ poor prognosis.
3. LINC01296 silencing suppressed HNSCC cells migration and invasion *in vitro*.
4. There were significant positive correlations between LINC01296 expression and TMB, MSI, MMRs.
5. The LINC01296 overexpression was related to promoter hypomethylation.
6. LINC01296 can be a good HNSCC diagnostic and prognostic biomarker.
